# From Genes to Proteins: The Indispensable Role of Proteogenomics in Advancing Clear Cell Renal Cell Carcinoma Research

**DOI:** 10.3390/ijms27115054

**Published:** 2026-06-03

**Authors:** Filip Kasperczak, Karolina Pawłowska-Kasperczak, Antoni Szuścik, Monika Zysnarska, Paweł Rajwa, Takafumi Yanagisawa, Fabio Zattoni, Michał Kasperczak

**Affiliations:** 1 Department of Urology, J. Struś Hospital in Poznań, Szwajcarska 3, 61-285 Poznan, Poland; filiperkasperczako@gmail.com (F.K.); kara040693@gmail.com (K.P.-K.); 2 Faculty of Medical Sciences, Prince Mieszko I Poznan Medical University of Applied Sciences, 60-320 Poznan, Poland; zysmonika@interia.pl; 3 Department of Urology, Comprehensive Cancer Center, Medical University of Vienna, 1090 Vienna, Austria; pawelgrajwa@gmail.com; 4 Second Department of Urology, Centre of Postgraduate Medical Education, 01-813 Warsaw, Poland; 5 Division of Surgery and Interventional Science, University College London, London NW3 2PS, UK; 6 Department of Urology, The Jikei University School of Medicine, Tokyo 105-8461, Japan; t.yanagisawa.jikei@gmail.com; 7 Department of Surgery, Oncology, and Gastroenterology, Urology Clinic, University of Padua, 35128 Padua, Italy; fabiozattoni@gmail.com

**Keywords:** clear cell renal cell carcinoma, proteogenomics, biomarkers

## Abstract

Clear cell renal cell carcinoma (ccRCC) is characterized by a complex molecular landscape driven by recurrent genetic alterations. While genomic and transcriptomic profiling have identified core drivers, they often fail to provide robust biomarkers due to the significant decoupling of mRNA and protein levels, as well as the critical role of post-translational modifications in tumor biology. This review synthesizes current evidence from landmark proteogenomic initiatives, such as the Clinical Proteomic Tumor Analysis Consortium (CPTAC), and independent multi-omic studies. It evaluates the integration of genomic, transcriptomic, and proteomic data to map metabolic reprogramming, signalling pathway activity, and chromatin-level alterations in ccRCC. Proteogenomic analyses reveal that protein-level data provide a functional perspective that is missing from sequencing alone, specifically identifying suppressed oxidative phosphorylation, enhanced glycolysis, and the activation of the PI3K/AKT/mTOR cascade, independent of genetic mutations. Furthermore, proteogenomics has defined novel molecular subtypes and individual protein biomarkers, such as UCHL1 and p-mTOR, which correlate more accurately with clinical outcomes and therapeutic responses than their transcriptomic counterparts. Proteogenomics is a crucial tool for refining disease taxonomy and identifying novel therapeutic vulnerabilities in ccRCC. By bridging the gap between genotype and functional phenotype, this integrated approach facilitates more precise risk stratification and accelerates the development of personalized medicine through better-informed selection of targeted and immune-based therapies.

## 1. Introduction

According to GLOBOCAN 2022, kidney cancer ranks as the 14th most frequently diagnosed malignancy worldwide [1]. Among them, renal cell carcinoma (RCC) accounts for approximately 85–90% of kidney cancers and is classified by the World Health Organization (WHO) into several histological subtypes: clear cell RCC (ccRCC) represents up to 70–90% of cases, followed by papillary RCC (10–15%) and chromophobe RCC (5–10%) [2,3,4]. At initial diagnosis, up to 30% of patients present with metastases, and approximately one-third will ultimately develop them [5]. Beyond established risk factors such as smoking, obesity, and hypertension, genetic alterations play a critical role in the pathogenesis of ccRCC [6]. The molecular landscape of ccRCC is highly complex, with pronounced intratumoral heterogeneity (ITH), as distinct genetic subclones and expression programs may coexist within the same tumor [7]. Early events include loss of the von Hippel–Lindau (*VHL*) tumor suppressor, which stabilizes hypoxia-inducible factors (HIFs) and drives angiogenic and metabolic reprogramming, while mutations in polybromo 1 (*PBRM1*), BRCA1-associated protein 1 (*BAP1*), and SET domain containing 2 (*SETD2*) further diversify tumor phenotypes [7,8].

Despite advances in defining the mutational landscape, genomic profiling alone has not been translated into robust prognostic or predictive biomarkers for clinical practice [9]. Transcriptomics, while enabling large-scale measurement of RNA abundance, does not capture the functional state of the tumor [7,10]. mRNA and protein levels are only weakly correlated in ccRCC, and transcript data provide no insight into post-translational modifications (PTMs) that govern protein activity, stability, and localization [10]. Consequently, integrative multi-omic approaches are increasingly applied to improve the understanding of tumor biology and to identify clinically relevant therapeutic targets. Among these, proteogenomics has gained particular attention as it directly links genomic and transcriptomic alterations with their protein-level consequences, including protein abundance, PTMs, protein–protein interactions, and pathway activity, thereby providing a functional perspective that complements genomics [11].

Although the Clinical Proteomic Tumor Analysis Consortium (CPTAC) and other large-scale proteogenomic initiatives have substantially expanded overall knowledge, the critical determinants of ccRCC biology and therapeutic response remain incompletely understood [12,13]. In particular, a comprehensive synthesis of proteogenomic findings in ccRCC remains lacking, one that integrates metabolic, signaling, and chromatin-level alterations with clinical phenotypes and delineates their implications for routine clinical practice. In this context, the present review synthesizes current evidence on the role of proteogenomics in ccRCC, emphasizing the limitations of genomic and transcriptomic profiling and the complementary functional information provided by proteomic analyses. It summarizes key insights into metabolic reprogramming, hypoxia signaling, and driver mutations, highlighting landmark studies and their clinical implications for biomarker discovery and therapy stratification. It also outlines remaining challenges and underscores the potential of proteogenomics to refine risk classification, identify therapeutic vulnerabilities, and accelerate clinical translation. To ensure a comprehensive and objective synthesis of the literature, a non-systematic literature search was conducted across PubMed, Scopus, and Web of Science databases for English-language articles published between 2010 and 2026. Search terms included ‘clear cell renal cell carcinoma’, ‘proteogenomics’, ‘mass spectrometry-based proteomics’, and ‘multi-omic integration’. Studies were selected based on their relevance to proteogenomic characterization, clinical biomarker validation, and therapeutic implications, with a specific focus on landmark consortium datasets (e.g., CPTAC) and independent multi-region profiling cohorts to minimize selection bias.

## 2. Why Genomics and Trascriptomics Alone Is Insufficient and How Proteomics Fills the Gaps in ccRCC Research

The genetic landscape of ccRCC is defined by recurrent alterations in key genes (*VHL*, *PBRM1*, *SETD2*, and *BAP1*), all of which are located on chromosome 3p and are summarized in Table 1. However, the reported mutation frequencies for these genes, along with their associated prognostic and predictive values, vary substantially between studies, a variance that reflects differences in patient cohort composition, sampling strategies, and sequencing platforms. Regarding *VHL* mutations, Huang et al. observed a prevalence of 59.7% in a Chinese patient cohort [14], Wang et al. reported a frequency of 67% [15], while Hu et al. described alterations in up to 90% of tumors [16]. This wide range should be interpreted cautiously, as individual studies differ in cohort composition, geographic background, tumor stage, sampling strategy, sequencing platform and analytical sensitivity. An additional source of variability is the definition of *VHL* alteration, particularly whether analyses capture only point mutations or also include copy-number loss and promoter methylation. Although *VHL* mutations are highly prevalent in ccRCC, they are not sufficient to initiate ccRCC, with their clinical impact largely dependent on co-occurring mutations in genes such as *PBRM1*, *SETD2*, and *BAP1* [17]. Understanding this gene interplay is critical, as *PBRM1* and *SETD2* mutations have been associated with both favorable and unfavorable outcomes across studies [18,19,20], whereas *BAP1* loss is generally linked to high-grade, aggressive disease but does not fully account for clinical heterogeneity [8]. These apparently conflicting associations should therefore be interpreted in a context-dependent manner, as the clinical effect of individual mutations is influenced by co-occurring genomic events, epigenetic regulation, tumor stage, treatment exposure and intratumoral heterogeneity. Multi-region sequencing has further revealed extensive ITH and complex patterns of co-mutation, with frequent co-occurrence of gene alterations within the same tumor [21,22]. Mechanistically, *PBRM1* deficiency amplifies HIF signaling, cooperating with *VHL* loss to promote tumorigenesis [20,23]. Interestingly, while *SETD2* mutations frequently co-occur with *PBRM1* alterations, this combination does not appear to confer a greater impact on prognosis than *PBRM1* loss alone [24]. In an exome-sequencing study, approximately half of *VHL*-mutant tumors also harbored *PBRM1* alterations. Moreover, all *SETD2*-mutant cases co-occurred with *PBRM1* or *VHL* mutations, and some tumors carried alterations in all three genes [25]. Taken together, these observations indicate that discrepancies between studies should not be viewed solely as inconsistencies, but as evidence that ccRCC biology is shaped by multilayered genomic architecture, spatial heterogeneity and context-dependent functional consequences of driver alterations. This provides a rationale for integrative proteogenomic approaches that move beyond single-gene interpretation and capture the downstream effects of genomic alterations at the protein and pathway levels.

Beyond inactivation of tumor-suppressor genes on chromosome 3p, ccRCC is characterised by recurrent arm-level copy number alterations (CNAs). Integrative genomic analyses have shown that, in addition to 3p loss, the most frequently deleted chromosomal arms include 14q (44–55%) and 9q (25%), whereas the most commonly amplified arms comprise 5q (49%), 7q (26%), 5p (24%), and 7p (24%) [31,32]. Such CNAs may modulate the expression of genes involved in chromatin regulation and growth-factor/angiogenic signalling (e.g., *SETD2*, *BAP1*, FLT4, PTEN, FGFR4, NSD1) and have been associated with advanced disease features and poor survival [33,34]. Deletions of 9p and 14q and gain of 7p are likewise significantly associated with features of advanced disease, such as tumor metastasis, T3/T4 stage, Fuhrman grade 3–4, larger tumor size, and poor outcome. Specifically, 9p loss, encompassing the tumor suppressor CDKN2A, correlates with increased translation initiation and activation of mTOR and MYC signalling, whereas 7p gain drives enhanced protein synthesis and epithelial–mesenchymal transition (EMT). Loss of 14q has been linked to a more aggressive clinical course, including distant metastases, reduced overall survival and an approximately threefold increased risk of death compared with patients lacking this alteration [33]. In particular, 14q loss affecting putative tumor suppressors NDRG2 (14q11.2) and HIF1A (14q23.2–q23.3) is associated with reduced WNT signalling and upregulation of MYC signalling, N-linked glycosylation and interferon-γ response [35]. Multivariate analyses further indicate that 5q gain increases the risk of large tumors, in line with proteogenomic evidence that 5q gain results in increased mTORC1 and MYC signalling [12]. Moreover, illustrative CNA-associated cis-cascades include SQSTM1 (5q35.3), OSBPL3 (7p15.3), and GOLPH3 (5q13.3), genes previously implicated in PI3K–mTOR pathway activity [12].

A primary limitation of relying on these genomic and transcriptomic structures is the targeted biological uncoupling between mRNA and protein levels, a phenomenon where ccRCC ranks among the lowest correlation coefficients across pan-cancer analyses (median Spearman *p* = 0.40–0.45). Quantitative profiling shows that while modern RNA-sequencing identifies 12,000 to 20,000 expressed transcripts, deep proteomics detects roughly 6000 to 10,000 proteins [12,35]. Rather than a global failure of translation, this discordance largely reflects technical limitations, strict analytical thresholds, and the presence of multiple regulatory layers. These layers include translational suppression by microRNAs, PTM, and altered signaling activity. Furthermore, metabolic rewiring and the tumor microenvironment reshape protein dynamics in ways that remain entirely invisible to transcriptomic analyses [10,36]. Pan-cancer analyses rank ccRCC among the tumor types with the lowest RNA–protein correlation [36,37]. Extending these findings, Qu et al. reported in a multi-omics cohort that only a subset of transcripts showed a measurable correlation with cognate proteins, while enzymes involved in central metabolism and hypoxia-responsive targets displayed particularly poor alignment [31]. From a translational perspective, low RNA–protein concordance implies that transcriptome-only biomarker strategies may overlook key protein determinants of tumor behavior or yield misleading candidates when RNA changes do not translate into functional protein alterations. For example, PD-L1 protein expression assessed by immunohistochemistry shows a more consistent association with therapeutic response than PD-L1 mRNA levels [38,39]. Consequently, effective therapeutic targeting of ccRCC requires systematic validation at the proteomic level, where pathway activity, protein abundance, and PTM status more accurately reflect the functional state of the tumor.

Transcript abundance reflects only the transcriptional potential of a cell, whereas proteins constitute the direct effectors of cellular physiology. This distinction is particularly relevant in ccRCC, where critical oncogenic and metabolic pathways are regulated predominantly through post-transcriptional and post-translational mechanisms [10]. A primary limitation of transcriptomic analysis in this context is the inability to detect protein stabilization; for instance, following *VHL* loss, HIF1A and EPAS1 (HIF2α) mRNA levels often remain unchanged, yet the proteins themselves accumulate and drive aggressive tumorigenesis. Furthermore, transcriptomic data often fail to capture the functional state of the tumor, underscoring the need for proteomic analyses to achieve accurate biological characterization. Similarly, phosphorylation-dependent activation of the mTOR and PI3K/AKT cascades highlights the central role of active kinase signaling captured exclusively through phosphoproteomic analyses [35,40]. Beyond abundance alterations, dysregulated PTM signatures control protein stability, localization, and interaction networks, thereby defining the disease’s functional architecture [41,42]. A recent proteomic study of early-stage ccRCC highlighted these dynamic shifts, including cytokine overproduction, hypersialylation, altered thiol/disulfide homeostasis, arginine hypermethylation, and stress-related protein modifications [41]. Together with alterations in soluble receptor expression and metabolic modulators, these PTM patterns provide high-resolution insight into tumor biology that can better inform biomarker development and therapeutic strategies. Ultimately, because transcriptome-only analysis lacks the resolution to capture these post-translational architectures, proteomics remains an indispensable tool for elucidating the true functional taxonomy of ccRCC.

## 3. Specific Benefits of Proteogenomics in ccRCC

Metabolic reprogramming is a central hallmark of ccRCC, shaped by genetic alterations and hypoxia-driven signaling [43,44]. Proteomic and metabolomic studies have demonstrated profound suppression of oxidative phosphorylation (OXPHOS) and remodeling of the TCA cycle, accompanied by enhanced glycolytic flux and lactate accumulation [45]. Beyond core energy production, ccRCC exhibits distinct alterations in the metabolism of specific molecules. For instance, the tumor acquires a dependence on glutamine, associated with the upregulation of GLS1, and displays dysregulated arginine metabolism linked to reduced ASS1 expression. Furthermore, lipid pathways are perturbed, showing impaired β-oxidation, cholesterol accumulation, and increased expression of lipogenic enzymes [43,46]. These metabolic shifts are fundamentally amplified by hypoxia-related signatures, which stabilize HIFs to drive extensive reprogramming across processes [47].

The PI3K/AKT/mTOR pathway is one of the most consistently dysregulated signaling axes in ccRCC, controlling cell survival, proliferation, metabolism, angiogenesis, and metastatic potential [48,49]. When *VHL* function is impaired, stabilized HIF-α subunits activate MAPK and AKT/mTOR cascades, thereby amplifying oncogenic signaling [50]. Phosphorylated mTOR (p-mTOR) serves as a functional marker of pathway activation, and high p-mTOR expression is strongly associated with adverse clinicopathological features [49]. Li et al. demonstrated a significantly elevated ratio of p-mTOR to total mTOR in tumor tissues compared to adjacent normal kidney, finding that increased levels correlated with larger tumor size, advanced pathological stage, metastasis, and reduced cancer-specific survival [49].

Alterations in PI3K/AKT/mTOR pathway genes occur only in a minority of ccRCCs but most commonly affect mechanistic target of rapamycin (MTOR), tuberous sclerosis complex 1 and 2 (TSC1, TSC2) and phosphatase and tensin homolog (PTEN). These mutations can nevertheless be functionally relevant for response to mTOR-targeted therapy. MTOR mutations are found in approximately 5–9% of tumors and are predominantly missense variants clustering in the FAT and kinase domains. Functional studies indicate that at least some of these variants can hyperactivate mTORC1 [51]. Other genetic events that activate mTORC1 include inactivating mutations or deletions of PTEN, which are likewise uncommon and occur in approximately 1–5% of ccRCCs. PTEN encodes a lipid and protein phosphatase that controls the balance between cell proliferation, apoptosis and migration through the PI3K/AKT/mTOR pathway [52]. Loss of PTEN function is expected to increase PIP3 levels, thereby promoting recruitment of PIP3-binding proteins, such as AKT isoforms, to the plasma membrane and enhancing PI3K/AKT/mTOR signalling [51,53]. In turn, AKT phosphorylates multiple substrates, including TSC2, which forms a tumor-suppressor complex with TSC1 [54]. Clinical studies suggest that TSC1 and TSC2 mutations may serve as predictive biomarkers of rapalog sensitivity in RCC [55]. Recent data also indicate that non-coding RNA networks may contribute to PI3K/AKT dysregulation in ccRCC. In particular, hsa-circ-0003596 has been shown to act as a competing endogenous RNA (ceRNA) for miR-502-5p, relieving repression of IGF1R and thereby activating PI3K/AKT signalling. Its overexpression enhances proliferation, invasion, and metastasis [56]. Moreover, the impact of mTOR extends to the tumor microenvironment, as its expression has been found to correlate with the infiltration of immune cell populations and immune checkpoint markers, such as PD-L1 [49]. Directly profiling these networks establishes a precise mapping of real-time pathway kinetics that correlates directly with clinical phenotypes.

**Figure 1:**
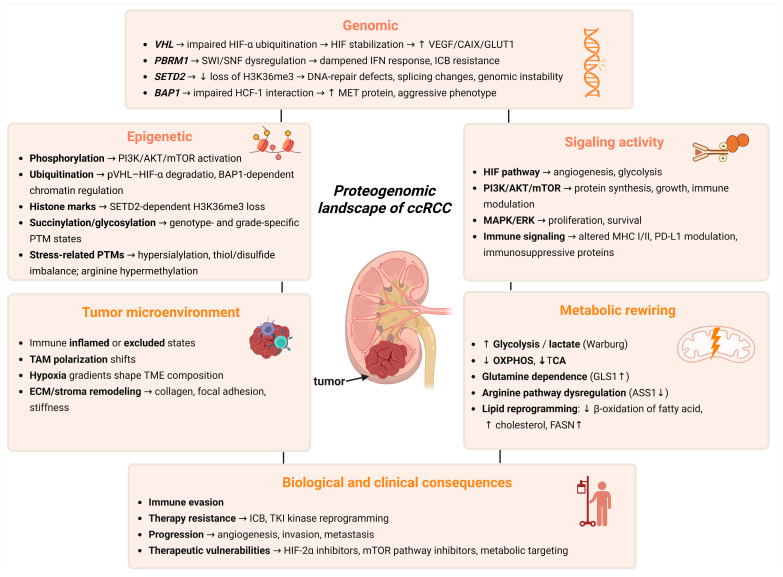
Multilayered alterations in ccRCC, including genomic, epigenetic, signaling, metabolic, and microenvironmental changes (arrows indicate direction of regulation: ↑ activation/overexpression, ↓ downregulation/suppression). Created in BioRender. Kasperczak, M. (2026) https://BioRender.com/4j3bg5l.

The complexity of ccRCC is further amplified by mutations in chromatin regulators, which reshape the PTMs of histones and associated proteins. For instance, *SETD2* loss leads to a marked reduction in the H3K36me3 and impaired chromatin integrity. Ho et al. demonstrated that, although *SETD2* mutations occur in early-stage primary tumors, the prevalence of H3K36me3 loss was substantially higher in advanced lesions [57]. This discrepancy, revealed by assessing the protein modification status, suggests the existence of non-genetic mechanisms capable of downregulating the mark. Recent proteogenomic profiling provided direct evidence that *BAP1* deficiency drives critical protein-level alterations with therapeutic implications. Specifically, Du et al. found that *BAP1*-mutant tumors display significantly increased c-Met protein abundance, establishing a clear link between chromatin-remodeling defects and the activation of pro-oncogenic MET signaling [58]. Moreover, the tumor-suppressive function of *BAP1* in ccRCC depends on its interaction with the transcriptional co-regulator HCF-1 [59]. Together, these findings highlight how convergent signalling alterations and chromatin-remodelling defects collectively shape the molecular landscape of ccRCC, as summarized in Figure 1.

The most direct benefit of integrating proteomic analysis is the systematic identification of clinically relevant protein biomarkers for ccRCC [60,61,62,63]. Tissue validation studies linked NDUFA4L2 (NADH dehydrogenase (ubiquinone) 1 alpha subcomplex, 4-like 2) protein expression to adverse pathological features but noted that its IHC staining did not consistently mirror transcript levels, highlighting the necessity of protein-level measurement [61]. Further extensive proteomic and IHC validation efforts have identified several key candidates. UCHL1 (Ubiquitin Carboxyl-Terminal Hydrolase L1) has been identified as a predictive biomarker; its significant upregulation was found in high-grade tumors and associated with poor prognosis [64,65]. However, the complex epigenetic control is underscored by contrasting reports that CpG hypermethylation of the UCHL1 promoter leads to reduced expression, which was likewise associated with adverse outcomes [66]. Another candidate is PAK4 (p21-activated kinase 4), which correlates with high Fuhrman grade, tumor necrosis, and shorter overall and recurrence-free survival [67].

Similarly, IHC evaluation confirmed proteomic findings that HEATR1 (HEAT Repeat Containing Protein 1) protein is upregulated, while SLC27A2 (Solute Carrier Family 27 Member 2) is downregulated in ccRCC; both alterations were associated with shorter progression-free survival (PFS) [68]. Consistently, Xu et al. confirmed that SLC27A2 expression is markedly reduced in ccRCC tissues, correlating with advanced pathological stage, presence of metastasis, and inferior survival outcomes [69]. Additional candidates include EFTUD2 (elongation factor Tu GTP binding domain containing 2), a spliceosomal factor that is significantly upregulated in ccRCC, and PROM1 (prominin-1/CD133), a transmembrane stem cell marker reported to be downregulated, with its expression suggesting regulation by hypoxia-driven pathways [70,71]. Finally, emerging evidence suggests that BCL9 (B-cell lymphoma 9), a co-activator of Wnt/β-catenin–dependent transcription, is consistently upregulated at the protein level in ccRCC and associated with shorter PFS [72,73]. In parallel, PD-L1 protein expression has been extensively investigated as a predictive biomarker for immune checkpoint inhibition in ccRCC. However, PD-L1 immunohistochemistry shows substantial intra-tumour heterogeneity and variable associations with treatment response across clinical trials [74].

From a clinical perspective, current proteomic studies identify a recurrent set of protein biomarkers consistently associated with more aggressive disease and poorer outcomes in ccRCC. High expression of UCHL1, PAK4, HEATR1, EFTUD2, BCL9, and PD-L1, together with low SLC27A2 and altered NDUFA4L2, has been associated across multiple cohorts with higher grade, advanced stage, metastasis, shorter progression-free survival, and shorter overall survival. To provide a clinically oriented overview, Table 2 summarises the most relevant protein biomarkers discussed in this review, together with their direction of change, reported clinical associations and current translational status. Although their predictive value remains exploratory, none of these biomarkers has been adopted by major international clinical guidelines, such as those of the European Association of Urology (EAU), the European Society for Medical Oncology (ESMO), or the National Comprehensive Cancer Network (NCCN), for routine risk stratification or treatment selection in ccRCC [75,76]. Current clinical practice still relies primarily on histopathological features and on integrated clinical prognostic scores, such as the Memorial Sloan Kettering Cancer Center (MSKCC) and International Metastatic RCC Database Consortium (IMDC) models, while protein-based biomarkers remain investigational [77]. Moving forward, implementation of these candidates in routine care will require validation in large, prospectively collected cohorts and standardisation of analytical assays. In addition, their clinical utility must be demonstrated formally by showing that they improve risk stratification and treatment selection beyond established prognostic models. Collectively, these efforts, particularly the integration of proteomic markers with genomic, transcriptomic, and microenvironmental features in biomarker-driven clinical trials, may enable the development of robust, clinically actionable classifiers for risk assessment and therapy selection in ccRCC.

## 4. Landmark Studies and Proteogenomic Insights in ccRCC

CPTAC has been a major driver of large-scale proteogenomic research, generating deeply integrated genomic, transcriptomic, and proteomic datasets in ccRCC that have provided key insights into metabolic remodeling, immune heterogeneity, and post-translational regulation (Table 3) [7,12,78]. Clark et al. provided the first comprehensive proteogenomic map of ccRCC, highlighting global decoupling of RNA and protein levels, extensive metabolic remodeling, and activation of oncogenic kinase signaling beyond what is evident from sequencing alone [12]. Building on this, Li et al. incorporated multi-region proteome, metabolome, and methylome data to map inter- and intratumor heterogeneity, relate proteomic patterns to immune contexture and genome instability, and delineate a molecularly defined high-risk subset with adverse outcomes [7]. Lih et al. added a glycoproteomic layer, demonstrating genotype- and grade-associated glycosylation patterns and their interplay with phosphorylation, thereby revealing an additional dimension of post-translational regulation in ccRCC [78].

Beyond consortium-led projects, CPTAC datasets have been widely reused by independent groups. Azuaje integrated proteomic and histopathological data, demonstrating that protein expression patterns align with tissue architecture and reflect immune-related processes, primarily driven by immune infiltration [79]. Chen et al. developed a machine learning-based prognostic model using CPTAC proteomics. This model, combining tumor size, grade, and stage, produced a risk score linked to proliferation-associated pathways (DNA repair, mitosis, cell cycle regulation) that significantly stratified patient survival [80]. Qiu et al. benchmarked patient-derived xenograft (PDX) models against CPTAC data, confirming that PDXs faithfully recapitulate subtype-specific proteomic features of advanced ccRCC and key pathways of progression and therapeutic resistance [81]. Additionally, independent studies have further elucidated ccRCC biology, focusing on metabolic and immune heterogeneity. Dong et al. performed integrated proteomic and lysine succinylation profiling, showing that global succinylation was markedly reduced in tumors, implicating the loss of this PTM in metabolic dysregulation [13]. Differentially succinylated proteins were predominantly mitochondrial, mapping to oxidative phosphorylation, the TCA cycle, and fatty acid metabolism [13]. Addressing ITH, Miheecheva et al. conducted multiregional single-cell proteogenomic analysis and found that cytokine signaling is a key driver of regional variation in the immune microenvironment [82]. Recent independent studies have further extended this framework by focusing on tumor-boundary biology, formalin-fixed paraffin-embedded (FFPE)-compatible proteomics, and spatial microenvironmental organisation. In *VHL* disease-associated ccRCC, Feilen et al. performed deep mass spectrometry-based proteomic profiling of FFPE samples and demonstrated that the tumor pseudocapsule represents a distinct ECM- and signalling-rich microenvironment rather than a purely structural boundary [83]. Similarly, Wess et al. integrated proteomics, scRNA-seq data, and multiplex imaging to identify ANXA4 as a ccRCC-enriched protein involved in membrane repair, invasion, and modulation of the tumor microenvironment [84]. These studies further support the value of independent, spatially informed proteomic approaches for capturing tumor–microenvironment interactions beyond bulk consortium datasets.

Integrated proteogenomics accelerates the translation of molecular findings into clinically actionable strategies in ccRCC by refining disease taxonomy with protein-level readouts, exposing druggable pathway activity, and generating biomarkers for prognosis, therapy prediction, and resistance. Understanding the *VHL*–HIF–VEGF axis has provided the rationale for therapeutic strategies targeting VEGF and, more recently, HIF-2α. The Phase III LITESPARK-005 trial demonstrated the superiority of Belzutifan, a first-in-class oral HIF-2α inhibitor, over everolimus, with a significant improvement in PFS and objective response rate [85]. Metabolic reprogramming is also a translational focus, where proteogenomic and metabolomic assays chart clinical dependencies across altered glycolytic pipelines and glutamine utilization networks [53]. These dependencies are not apparent at the transcriptomic level. The clinical evaluation of pharmacological inhibition of glutamine metabolism, such as the combination of nivolumab with the glutaminase inhibitor telaglenastat, is currently ongoing, though definitive efficacy remains to be established [86]. Parallel advances have also emerged from therapeutic targeting of the VEGF/VEGFR signaling axis. The central role of VEGF-driven angiogenesis in ccRCC progression provided the rationale for the development of VEGFR tyrosine kinase inhibitors (VEGFR-TKIs), which remain a cornerstone of systemic therapy [87,88]. More recently, proteogenomic insights into the interplay between angiogenic and immune signaling have supported the integration of VEGFR-TKIs with immune checkpoint inhibitors. In support of this approach, randomized phase III trials, including KEYNOTE-426 (pembrolizumab plus axitinib) and CheckMate-9ER (nivolumab plus cabozantinib), have demonstrated significant improvements in both progression-free and overall survival [89,90]. Collectively, these observations underscore the expanding translational opportunities arising from proteogenomic insights in ccRCC, while emphasizing that the clinical efficacy of many emerging targeted and combination strategies remains to be fully established. To bridge the gap between high-dimensional omics and the clinic, proteogenomic subtypes can be distilled into minimal IHC panels featuring markers such as *BAP1*, *PBRM1*, and UCHL1, providing a rapid surrogate for genomic instability and aggressive disease [7]. These protein-level readouts offer a “molecular compass” for therapy selection: *PBRM1*-deficient tumors often exhibit a strong angiogenic signature favoring VEGFR-TKI therapy [91], whereas *BAP1*-deficient tumors with high immune contexture may be better suited for immune checkpoint inhibitor combinations [92]. By focusing on these functional protein expressions rather than just DNA mutations, clinicians can more accurately match the specific pathway activation of a tumor to the most effective targeted or combination treatment.

## 5. Clinical Implications: Biomarkers, Subtypes, and Therapy

Proteogenomics significantly enhances the clinical management of ccRCC by providing a more comprehensive understanding of the tumor’s biology compared to genomics alone. This integrated approach enhances risk stratification, informs therapeutic decisions, and facilitates the identification of novel biomarkers. It refines prognosis by identifying distinct molecular subtypes with different clinical outcomes. While traditional staging systems are helpful, they often fail to capture the full aggressiveness of a tumor. A CPTAC study used multi-omics types to define subtypes: proteomics defined four subtypes (e.g., CD8(+) inflammatory, immune-deserted metabolic), phosphoproteomics found four distinct subtypes (P1–P4) with different tumor grades, and glycoproteomics identified three subtypes (Glyco1–3) [7]. Integrating these data streams yielded three final multi-omic subtypes (NMF1–NMF3). Crucially, NMF1 was linked to a worse prognosis and a higher hazard ratio. Extending these observations, Hu et al. integrated multi-omics with single-cell and spatial analyses to delineate four disease subtypes, including a de-differentiated clear cell (DCCD) [86]. Importantly, DCCD emerged as a common trajectory of ccRCC progression and was associated with poor outcomes and early recurrence, even at stage I. Additionally, they identified four immune subtypes (IM1–IM4). A proteogenomic analysis of a Chinese ccRCC cohort classified ccRCC into three proteomic subtypes (GP1–3) [31]. GP1 was identified as an aggressive subtype resembling the CPTAC CD8+ inflamed tumors. The subtype exhibited a strong immune phenotype (T-cell and macrophage protein upregulation) alongside strong immunosuppressive, matrix remodeling, and angiogenic signatures, resulting in increased metastatic potential and poor prognosis. In contrast, GP2 was characterized by high tumor purity and exhibited broad metabolic activation, involving the TCA cycle, amino acid and lipid metabolism, and glycolysis/gluconeogenesis, which collectively define a metabolic immune-desert phenotype. GP3 was distinct, exhibiting high stromal content and strong extracellular matrix (ECM) signatures, coherent with an immune-excluded microenvironment [31]. Miranda-Poma et al. performed DIA-proteomics and identified two distinct proteomic subtypes, CC1 and CC2, which could not be resolved by genomic or transcriptomic analyses [93]. CC1 was characterized by proteins related to mitochondrial metabolism, glycolysis, ribosomal translation, and cytoskeletal adhesion, reflecting a metabolically active and translationally engaged phenotype. In contrast, CC2 exhibited increased expression of ECM and stromal components, including pathways for focal adhesion and collagen organization, indicative of a microenvironmentally driven program [93]. This two-subtype classification is consistent across different studies, although it shows varying survival rates [12].

These stratification systems demonstrate the ability of protegenomic data to classify ccRCC into biologically distinct entities. Next-generation sequencing often misses crucial protein modifications that reveal a tumor’s biological state, such as PTMs like phosphorylation and glycosylation [10]. These PTMs are essential for regulating key cellular processes, including adhesion, migration, and signaling, and contribute to the metabolic shifts observed in ccRCC [94,95,96,97]. Despite over a decade of research, a complete proteomic profile that links genomic aberrations to clinical outcomes remains an unfulfilled goal. Nevertheless, proteomic analyses have already enabled the categorization of ccRCC patients, helping to distinguish those who might benefit from VEGF inhibitors versus immune-based therapies [12,98]. Targeted therapies such as VEGF and mTOR inhibitors were developed based on an understanding of ccRCC biology [99,100,101,102,103]. The response to treatment is often linked to specific mutations and protein signatures. *PBRM1*-mutated tumors responded well to anti-angiogenic therapy in the RECORD-3 and IMmotion 150 studies [104,105]. However, the IMmotion 150 study also suggested a worse outcome with PD-L1 blocking therapy [105]. Conflicting reports exist on how *PBRM1* mutations affect the response to checkpoint inhibitors, with one study associating them with a better response [106,107], while a murine model showed *PBRM1* loss reduced immune infiltration and increased resistance to anti-PD-1 antibodies [108]. In the JAVELIN trial, IM1, IM3, and IM4 (DCCD-ccRCC) subtypes, when treated with avelumab (PD-L1 inhibitor) and axitinib (VEGFR inhibitor), showed improved PFS over sunitinib [86,109]. Conversely, IM2 patients did not show a similar benefit from this combination therapy in both the JAVELIN and IMmotion 151 trials [86,110]. The CheckMate cohort found that treatment with nivolumab, a PD-1 inhibitor, extended OS in IM2 and IM3 patients over everolimus, an mTOR inhibitor [86,111].

Several individual proteins have been identified as key prognostic markers. High expression of UCHL1 is associated with poor survival, *BAP1* mutations, and genomic instability [7]. A protein signature score using the abundance of LRRC59, RPN2, and SERPINH1 also serves as a prognostic indicator [7]. The AXL protein, whose gene expression is associated with aggressive ccRCC, has levels that increase following sunitinib treatment [112,113]. In a 2021 study, a panel of secreted proteins was found to be highly expressed in ccRCC tissues, suggesting potential non-invasive markers, including SPARC [114]. Another study found that high expression of SERPINA3 was linked to poor prognosis, with its abundance increasing in metastatic tissue, indicating its role in advanced, treatment-resistant disease [115].

**Figure 2:**
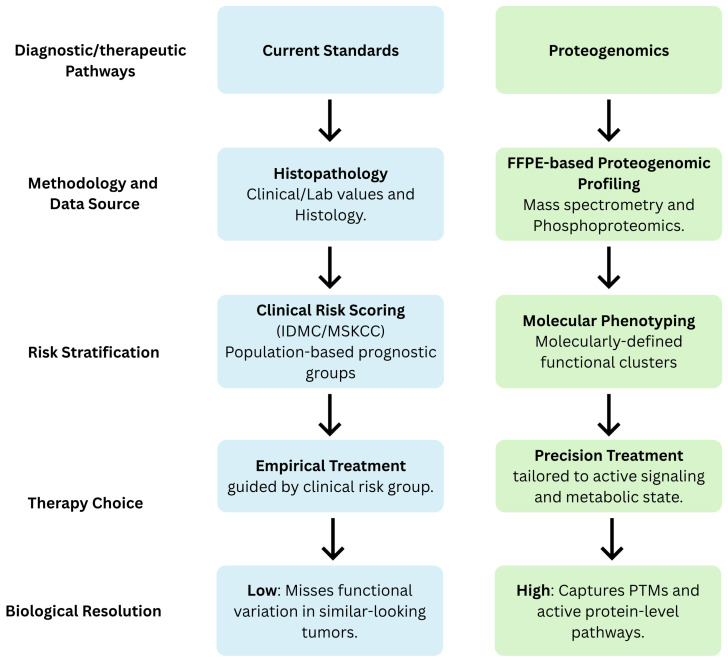
Comparative Analysis of Diagnostic and Therapeutic Pathways in ccRCC. The standard pathway relies on systemic clinical parameters and histopathology for empirical treatment selection. The proteogenomic pathway utilizes functional molecular phenotyping, including protein abundance and post-translational modifications (PTMs), to transition from population-based risk stratification to individualized precision medicine. Proteogenomics is intended to complement rather than replace current standards.

Beyond established genomic subtypes, proteomic analysis uncovers functional differences in metabolic and signaling pathways. These proteomic subtypes, which can be independent of genomic drivers, offer a new way to classify tumors based on their active biology. This deeper understanding of the tumor’s functional state allows for the development of more precise therapies and the identification of new biomarkers for diagnosis and treatment monitoring. To better contextualize how these molecular findings improve upon existing pathways, Figure 2 illustrates the shift from a reactive to a proactive diagnostic framework. While current clinical standards are robust for population-level prognosis, they lack the granularity to predict individual therapeutic sensitivity. An integrated proteogenomic pathway complements these models by providing a ‘functional layer’, for example, distinguishing between ‘Angiogenic-High’ and ‘Immune-Inflamed’ phenotypes, thereby allowing treatment selection to be guided by the tumor’s active biology rather than statistical probability.

## 6. Challenges, Limitations, and Future Directions in Clinical Proteogenomics

**Figure 3:**
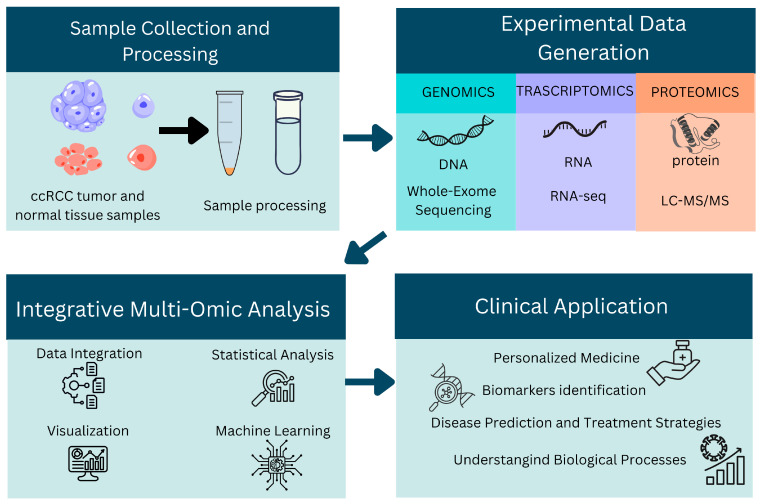
Proteogenomic Integration Workflow in ccRCC. The process moves from Sample Collection of tumor and normal tissues to Experimental Data Generation across genomic (DNA), transcriptomic (RNA), and proteomic (protein) layers. These datasets are unified through Integrative Analysis using statistical and computational tools to bridge the gap between genetic blueprints and functional phenotypes. This synthesis enables Clinical Applications, including biomarker discovery, disease prediction, and the advancement of personalized treatment strategies.

Despite its great potential, the widespread clinical application of proteogenomics faces several technical and logistical challenges that must be overcome for its successful translation into clinical practice [116]. The field is rapidly advancing, with emerging technologies poised to revolutionize the diagnosis and treatment of conditions like ccRCC by overcoming limitations and heading towards personalized medicine (Figure 3).

One of the primary difficulties is the technical limitations inherent to proteomics, particularly concerning its dynamic range, reproducibility, and tissue requirements. The vast complexity of the human proteome and the wide range of protein concentrations create a major analytical challenge, where highly abundant proteins obscure rarer ones, complicating quantitative analysis and biomarker discovery [117]. Accurate protein quantification is difficult due to variability and a general lack of standardization across methods hinders reliable data [118]. Additionally, proteomic studies rely on invasive tissue sampling, which raises ethical concerns and tissue processing to alter protein structures during processing [119,120,121,122,123]. Furthermore, current methods struggle to capture ITH due to limited spatial resolution [84]. In addition to analytical limitations, proteomic measurements are highly sensitive to pre-analytical variables, including tissue handling, cold ischaemia time, fixation conditions, tumor purity, and spatial sampling. These factors are particularly relevant in ccRCC, where marked intratumoral heterogeneity means that a single biopsy or tumor region may not fully represent the molecular landscape of the disease. Reproducibility may also be affected by platform-dependent differences in mass-spectrometry acquisition, sample preparation, peptide fractionation, batch correction, normalisation and data-processing pipelines.

Integrating data from multiple ‘omics’ layers is also complex. Genomics does not fully capture protein interactions or PTMs [124,125,126], and the correlation between mRNA and protein levels is often partial [127,128,129]. Additionally, the link between genetic variation and the proteome is non-linear [130], requiring sophisticated functional interpretation. This analysis is hampered by a fragmented computational landscape, where a lack of standardized software leads to inconsistencies [118,131]. Beyond these technical hurdles, the shift from discovery to routine practice hinges on significant practical and economic barriers. High-dimensional proteogenomic assays currently require expensive, specialized infrastructure that is largely restricted to academic centers, making advanced proteogenomic experiments expensive and time-consuming [132]. For these techniques to achieve clinical utility, turnaround times must be reduced to align with standard decision-making windows [133]. Targeted proteomics, with quick analysis times and high reproducibility, is identified as the only viable pathway for clinical translation compared to discovery-based methods, which require longer per-sample times and suffer from significant inter-laboratory variability [134,135]. Furthermore, reimbursement remains a major obstacle; health systems and insurers require prospective evidence that proteomic stratification improves patient outcomes or reduces the costs associated with ineffective standard-of-care treatments. Accordingly, clinical implementation will require analytically validated assays, cross-cohort reproducibility, standardised workflows and prospective evidence that proteomic stratification improves risk assessment or treatment selection beyond established clinical models. Ultimately, standardizing these functional tests will be essential for moving proteogenomics from a research-intensive endeavor to a sustainable component of precision oncology.

Looking ahead, proteogenomics is poised to revolutionize patient stratification and tailor therapeutic strategies by providing a comprehensive understanding of tumor phenotypes [10] matching therapies to an individual’s unique molecular profile [136]. The field also offers a powerful, non-invasive platform for identifying and quantifying urinary biomarkers for earlier disease detection [114,137]. New technologies are emerging to address ITH [116]. Single-cell proteogenomics aims to quantify proteins at the cellular level [82]. Managing the resulting enormous datasets requires significant technological solutions, including the seamless integration of multi-omics data [138,139]. Standardization and collaboration are key, with organizations like CPTAC, TCGA, and ICPC actively working to establish unified proteogenomic pipelines [140]. Finally, the use of advanced computational tools, including efficient algorithms and high-performance computing, is essential. Machine learning is playing an increasingly critical role in interpreting massive datasets and identifying subtle patterns [141]. This integration of multi-omics data, driven by next-generation technologies, will make proteogenomics an indispensable tool for personalized medicine.

While the discovery phase of proteogenomics has yielded thousands of candidates, clinical translation requires a structured prioritization based on cross-cohort reproducibility and compatibility with standard diagnostic workflows, such as Formalin-Fixed Paraffin-Embedded (FFPE) tissue analysis. The integration of these data provides a functional layer of insight that genomic profiling alone cannot capture, particularly in ccRCC where the correlation between mRNA and protein abundance is weak [142]. Promising biomarkers, such as p-mTOR, reflect the tumor’s signaling state rather than just its genetic potential, and show high reproducibility across diverse populations [12,143]. Furthermore, protein-level assessments of *BAP1* and *PBRM1* status provide a reliable framework for risk stratification [144,145], and metabolic markers such as SLC27A2, HEATR1, and NNMT have demonstrated clinical feasibility through their compatibility with standard immunohistochemistry and stability during formalin fixation [68,146]. Recent advancements in micro-scaled proteogenomics have even enabled high-resolution mass spectrometry on single-slide FFPE sections, facilitating the retrospective analysis of vast clinical archives [147]. Despite these strides, including the recognition of KIM-1 as a potent circulating biomarker for recurrence (NCT03024996) [148], transition into formal guidelines like the EAU or NCCN requires definitive proof that these signatures improve treatment selection beyond current clinical models, ultimately leading to a refined disease taxonomy that distinguishes patients who benefit from anti-angiogenic therapies versus immune checkpoint inhibitors.

The clinical transition of proteogenomics is evidenced by several prospective trials moving the “functional layer” from discovery to validation. KIM-1 is currently being validated as a routine biomarker (NCT01063998), while studies on urinary extracellular vesicles (EVs) (NCT07243067) and bone morphogenetic protein antagonist-regulated cancer (BARC) levels (NCT00900276) evaluate the feasibility of non-invasive diagnostics. To refine therapy selection, the NCT06211790 trial prospectively stratifies metastatic ccRCC patients into three treatment arms (GP1–GP3) to compare targeted versus immune-based efficacy. Predictive biomarkers are also being explored through phosphorylation profiling to predict TKI response (NCT02071719) and through mass spectrometry-based analysis of urinary metabolites and proteins (NCT04712305) to forecast immunotherapy effectiveness and toxicity. Further integration of multi-omic data (NCT07038733) and innovative imaging, such as the INTROSPECTION study (NCT06976021), which combines blood proteomics with Immuno-PET, highlights the move toward real-time treatment monitoring. Finally, the Phase IV NCT06903312 trial investigating primary tumor intervention in immunotherapy responders provides a prospective resource for identifying future tumor-specific biomarkers. These trials represent the critical next step in moving proteogenomics from the laboratory to the bedside.

## 7. Conclusions

In conclusion, the trajectory of clinical proteogenomics in ccRCC is moving from a phase of high-dimensional discovery to one of pragmatic, evidence-based application. While technical barriers such as analytical standardization and the complexity of multi-omic integration remain significant, the emergence of FFPE-compatible assays and non-invasive testing is rapidly lowering the threshold for clinical entry. The success of ongoing prospective trials will be the final arbiter of this transition, providing the level of evidence required to incorporate proteomic signatures into international treatment guidelines. By shifting the diagnostic focus from a tumor’s genetic potential to its actual functional state, these proteomic signatures will enable a refined disease taxonomy.

## Figures and Tables

**Table 1:** Frequently altered genes in ccRCC and their functional and clinical relevance; * ICB: immune checkpoint blockade.

Gene	Chromosome	Protein	Mutation Frequency (%)	Function	Clinical Associations	References
*VHL*	3p25-26	pVHL	45–90	E3 ubiquitin ligase regulating HIFα degradation	Initiation of tumorigenesis; promotion of angiogenesis	[9,26,27]
*PBRM1*	3p21	Polybromo 1	30–41	Component of the SWI/SNF complex	Modulation of the immune microenvironment; potential enhancement of response to ICB *	[24,25,28]
*SETD2*	3p21	SET domain-containing 2	8–30	H3K36 methyltransferase; modulates Wnt/β-catenin signaling	Genomic instability; aggressive clinicopathological behavior	[9,29]
*BAP1*	3p21	BRCA1-associated protein 1	6–19	Nuclear deubiquitinase	High histological grade; poor survival	[9,30]

**Table 2:** Key protein biomarkers in ccRCC and their clinical relevance.

Biomarker	Main Alteration	Clinical Relevance
p-mTOR	Increased phosphorylation/increased p-mTOR to total mTOR ratio	Associated with larger tumor size, advanced pathological stage, metastasis and reduced cancer-specific survival;
NDUFA4L2	Altered protein expression; IHC not consistently concordant with transcript levels	Associated with adverse pathological features, including lymphovascular invasion and higher pathological tumor stage
UCHL1	Increased protein expression; promoter hypermethylation-associated reduced expression has also been reported	Associated with high-grade tumors, genomic instability and poor prognosis; epigenetic silencing also linked to adverse outcome
PAK4	Increased protein expression	Associated with high Fuhrman grade, tumor necrosis, shorter overall survival and shorter recurrence-free survival
HEATR1	Increased protein expression	Associated with shorter progression-free survival
SLC27A2	Reduced protein expression	Associated with shorter progression-free survival, advanced pathological stage, metastasis, and inferior survival outcomes
EFTUD2	Increased protein expression	Associated with progression-free survival
PROM1	Reduced expression	Expression linked to hypoxia-related pathways
BCL9	Increased protein expression	Associated with shorter progression-free survival and adverse clinical features
PD-L1	Increased protein expression	Extensively studied as a predictive biomarker for immune checkpoint inhibition; affected by intra-tumor heterogeneity and variable association with treatment response
*BAP1*/*PBRM1*	Protein-level loss or altered expression	Supports risk stratification and may reflect aggressive biology or therapy-relevant tumor states
AXL	Increased protein level	Associated with aggressive ccRCC biology and sunitinib resistance
SERPINA3	Increased protein expression	Associated with poor prognosis; increased abundance in metastatic tissue suggests relevance to advanced and treatment-resistant disease
KIM-1	Increased circulating and/or urinary protein levels	Recognised as a potential circulating biomarker for recurrence; under prospective clinical validation

**Table 3:** Major CPTAC proteogenomic studies in ccRCC.

Study	Cohort/Data	Methods	Key Findings
Clark et al. [12]	110 ccRCC tumors, 84 normal adjacent tissue	Proteome, phosphoproteome (whole-exome sequencing, RNA-seq)	Weak mRNA–protein concordance; downregulation of OXPHOS, fatty-acid metabolism, and TCA-cycle proteins; enrichment of hypoxia, glycolysis, immune and EMT programs; mTOR/MAPK hyperactivation converging on EIF4EBP1 phosphorylation
Li et al. [7]	213 multi-region ccRCC samples	Proteome, phosphoproteome, metabolome, methylome	Defined inter- and intratumoral heterogeneity; ~50% of tumors with spatially divergent immune/endothelial signatures; identified a high-risk subset (high grade, *BAP1* loss, genomic instability, DNA hypermethylation); *UCHL1* identified as a marker of aggressive disease; accumulation of glutamine and urea-cycle intermediates in high-grade tumors
Lih et al. [78]	103 ccRCC tumors, 80 normal adjacent tissue	Glycoproteomics + proteomics	Subtype-specific glycosylation patterns: *BAP1*-mutant tumors enriched in high-mannose and sialylated glycans, and *PBRM1*-mutant tumors enriched in fucosylated glycans; grade-associated shift from sialylation in low-grade to oligomannose in high-grade tumors; evidence for glycosylation–phosphorylation crosstalk

## Data Availability

No new data were created or analyzed in this study. Data sharing is not applicable to this article.
